# Left atrial structure and function are associated with cardiovascular outcomes independent of left ventricular measures: a UK Biobank CMR study^[Author-notes jeab266-FM2]^

**DOI:** 10.1093/ehjci/jeab266

**Published:** 2021-12-15

**Authors:** Zahra Raisi-Estabragh, Celeste McCracken, Dorina Condurache, Nay Aung, Jose D Vargas, Hafiz Naderi, Patricia B Munroe, Stefan Neubauer, Nicholas C Harvey, Steffen E Petersen

**Affiliations:** William Harvey Research Institute, NIHR Barts Biomedical Research Centre, Queen Mary University of London, Charterhouse Square, London EC1M 6BQ, UK; Barts Heart Centre, St Bartholomew’s Hospital, Barts Health NHS Trust, London EC1A 7BE, UK; Division of Cardiovascular Medicine, Radcliffe Department of Medicine, University of Oxford, National Institute for Health Research Oxford Biomedical Research Centre, Oxford University Hospitals NHS Foundation Trust, Oxford OX3 9DU, UK; London North West University Healthcare NHS Trust, Harrow HA1 3UJ, UK; William Harvey Research Institute, NIHR Barts Biomedical Research Centre, Queen Mary University of London, Charterhouse Square, London EC1M 6BQ, UK; Barts Heart Centre, St Bartholomew’s Hospital, Barts Health NHS Trust, London EC1A 7BE, UK; William Harvey Research Institute, NIHR Barts Biomedical Research Centre, Queen Mary University of London, Charterhouse Square, London EC1M 6BQ, UK; MedStar Georgetown University Hospital, Washington, DC 20007, USA; William Harvey Research Institute, NIHR Barts Biomedical Research Centre, Queen Mary University of London, Charterhouse Square, London EC1M 6BQ, UK; Barts Heart Centre, St Bartholomew’s Hospital, Barts Health NHS Trust, London EC1A 7BE, UK; William Harvey Research Institute, NIHR Barts Biomedical Research Centre, Queen Mary University of London, Charterhouse Square, London EC1M 6BQ, UK; Division of Cardiovascular Medicine, Radcliffe Department of Medicine, University of Oxford, National Institute for Health Research Oxford Biomedical Research Centre, Oxford University Hospitals NHS Foundation Trust, Oxford OX3 9DU, UK; MRC Lifecourse Epidemiology Unit, University of Southampton, Southampton, UK; NIHR Southampton Biomedical Research Centre, University of Southampton, University Hospital Southampton NHS Foundation Trust, Southampton, UK; William Harvey Research Institute, NIHR Barts Biomedical Research Centre, Queen Mary University of London, Charterhouse Square, London EC1M 6BQ, UK; Barts Heart Centre, St Bartholomew’s Hospital, Barts Health NHS Trust, London EC1A 7BE, UK; Health Data Research UK, London, UK; Alan Turing Institute, London, UK

**Keywords:** lef, t atrium, left ventricle, cardiovascular magnetic resonance, vascular risk factors, atrial fibrillation, stroke, ischaemic heart disease, cardiovascular outcomes, mortality

## Abstract

**Aims:**

We evaluated the associations of left atrial (LA) structure and function with prevalent and incident cardiovascular disease (CVD), independent of left ventricular (LV) metrics, in 25 896 UK Biobank participants.

**Methods and results:**

We estimated the association of cardiovascular magnetic resonance (CMR) metrics [LA maximum volume (LAV), LA ejection fraction (LAEF), LV mass : LV end-diastolic volume ratio (LVM : LVEDV), global longitudinal strain, and LV global function index (LVGFI)] with vascular risk factors (hypertension, diabetes, high cholesterol, and smoking), prevalent and incident CVDs [atrial fibrillation (AF), stroke, ischaemic heart disease (IHD), myocardial infarction], all-cause mortality, and CVD mortality. We created uncorrelated CMR variables using orthogonal principal component analysis rotation. All five CMR metrics were simultaneously entered into multivariable regression models adjusted for sex, age, ethnicity, deprivation, education, body size, and physical activity. Lower LAEF was associated with diabetes, smoking, and all the prevalent and incident CVDs. Diabetes, smoking, and high cholesterol were associated with smaller LAV. Hypertension, IHD, AF (incident and prevalent), incident stroke, and CVD mortality were associated with larger LAV. LV and LA metrics were both independently informative in associations with prevalent disease, however LAEF showed the most consistent associations with incident CVDs. Lower LVGFI was associated with greater all-cause and CVD mortality. In secondary analyses, compared with LVGFI, LV ejection fraction showed similar but less consistent disease associations.

**Conclusion:**

LA structure and function measures (LAEF and LAV) demonstrate significant associations with key prevalent and incident cardiovascular outcomes, independent of LV metrics. These measures have potential clinical utility for disease discrimination and outcome prediction.

## Graphical Abstract

**Graphical Abstract:**
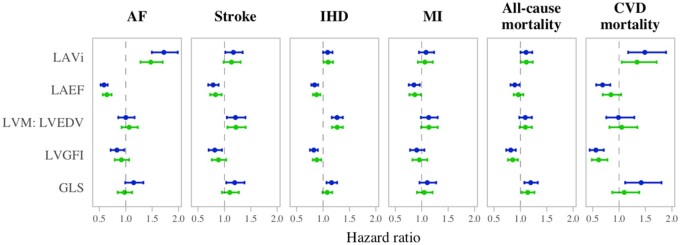
Association of CMR metrics with incident cardiovascular disease considered separately (blue) and in models mutually adjusted for all the CMR metrics (green). The points indicate point estimate for the hazard ratio and the intervals indicate the corresponding 95% confidence intervals from Cox hazard proportional models. Confounders are age, sex, ethnicity, deprivation, education, body mass index, hypertension, high cholesterol, diabetes, physical activity, and smoking. The blue results are from models with individual adjustment for CMR metrics (Supplementary data online, *Table S9*). The green results are from models mutually adjusting for all the CMR metrics (*Table 3*). AF, atrial fibrillation; CMR, cardiovascular magnetic resonance; CVD, cardiovascular disease; LVGFI, left ventricular global function index; GLS, global longitudinal strain; i, indicates indexation to body surface area; IHD, ischaemic heart disease; LAEF, left atrial ejection fraction; LAV, maximum left atrial volume; LVEDV, left ventricular end-diastolic volume; LVM, left ventricular mass; MI, myocardial infarction.

## Introduction

The left atrium (LA) is highly sensitive to subtle left ventricular (LV) haemodynamic changes.^1,^^2^ Alterations in LA structure and function may precede detectable LV dysfunction and, as such, have potential utility for earlier and more accurate disease discrimination than LV metrics.^1–3^ In particular, LA size and function are altered in response to elevated LV filling pressures, an early feature of diastolic dysfunction and a key component of heart failure with preserved ejection fraction (HFpEF).^1,^^3^ Furthermore, clinically important arrhythmias, such as atrial fibrillation (AF), primarily result in atrial (rather than ventricular) remodelling. Thus, atrial metrics may provide better indicators for the presence and occurrence of these conditions and provide incremental predictive value for key related health outcomes, such as stroke.^4^

The association of echocardiography derived measures of LA structure and function with incident and prevalent cardiovascular diseases (CVDs) has been repeatedly demonstrated.^5–9^ However, whilst the incremental value of LA over LV metrics seems biologically plausible, formal demonstration of this requires further study. Furthermore, although echocardiography is a valuable first line modality in clinical settings, cardiovascular magnetic resonance (CMR) is the reference standard for cardiac chamber quantification providing highly reproducible metrics calculated with fewer geometric assumptions than in echocardiography. Existing CMR studies of the utility of LA metrics are mostly based on small select samples of clinical cohorts,^10–12^ with a paucity of data from larger population-based samples.

The UK Biobank is a very large population-based cohort study including detailed participant characterization, linked longitudinally tracked health outcome data, and detailed standardized CMR. Thus, we evaluated, in 25 896 UK Biobank participants, clinical associations of LA structure and function independent of LV metrics. We estimated associations of CMR derived LA and LV metrics with vascular risk factors (VRFs), prevalent CVD, incident CVD, and mortality outcomes. We considered a wide range of demographic and clinical confounders and, critically, we assessed the independent value of LA metrics over measures of LV structure and function.

## Methods

### Setting and study participants

The UK Biobank includes over 500 000 participants from across the UK. Individuals aged 40–69 years old were identified using National Health Service (NHS) registers and recruited between 2006 and 2010 through postal invitations.^13^ Baseline assessment comprised detailed characterization of participant demographic, lifestyle, environmental, and medical factors, as well as a series of physical measures and blood sampling. Individuals who could not complete baseline assessment due to discomfort or ill health were not recruited. The UK Biobank protocol is publicly available.^14^ Linkages have been established with key routine health data including hospital episode statistics (HES) and death registers, with health outcomes documented according to standardized International Classification of Diseases (ICD) codes. This linked information is continually updated allowing reliable longitudinal tracking of incident events for all participants. Furthermore, the UK Biobank has produced adjudicated algorithmically defined incident health outcome data for key illnesses, such as myocardial infarction (MI) and stroke.^15^ The UK Biobank imaging study, launched in 2015, aims to scan a random 100 000 subset of the original participants and includes, amongst other things, detailed CMR imaging.^16^

### CMR image acquisition

The UK Biobank imaging study is performed using standardized pre-defined operating procedures, equipment, and staff training. CMR imaging was with 1.5 T scanners (MAGNETOM Aera, Syngo Platform VD13A, Siemens Healthcare, Erlangen, Germany), the acquisition protocol is published elsewhere.^17^ In brief, cardiac function assessment comprised three long axis cines and a complete short axis stack covering the left and right ventricles acquired at one slice per breath hold using balanced steady-state free precession sequences.

### CMR image analysis

CMR indices were derived using a fully automated quality-controlled image analysis pipeline previously developed and validated in a large subset of the UK Biobank.^18,^^19^ CMR metrics were available for the first 26, 891 UK Biobank CMR studies, of these both LA and LV data were available for 25 896 participants, which we include in the study (Supplementary data online, *Figure S1*). We considered the following CMR measures: LA maximum volume (LAV), LA ejection fraction (LAEF, calculated as: LA maximum volume−LA minimum volume/LA maximum volume), LV mass : LV end-diastolic volume ratio (LVM : LVEDV), and global longitudinal strain (GLS). We considered LV global function index (LVGFI) as an additional measure of LV function. Previous reports have identified LVGFI as a strong predictor of heart failure and CVD events with incremental utility over LV ejection fraction (LVEF).^20,^^21^ As per previous descriptions,^20,^^21^ we defined LVGFI (%) as LV stroke volume/LV global volume × 100, where LV global volume was calculated as the sum of the LV mean cavity volume [(LV end-diastolic volume + LV end-systolic volume)/2] and myocardium volume (LV mass/density). Density of LV was specified as 1.05 g/mL. A higher LVGFI reflects better LV function. As LVEF is a more clinically established metric, we also considered associations with LVEF and compared its performance to LVGFI.

### Defining participant characteristics

Sex and ethnicity were taken as self-reported at baseline. Ethnicity was converted into a binary variable of White and Black Asian and minority ethnic (BAME) groups. Socioeconomic deprivation was recorded at the baseline UK Biobank assessment as the Townsend index, a measure of deprivation relative to national averages.^22^ Age was calculated at the time of imaging. Body mass index (BMI) was calculated from height and weight recorded at imaging. Educational level and smoking status were taken from self-report. Physical activity level was expressed as a continuous value of metabolic equivalent (MET) minutes/week, calculated by weighting different types of activity (walking, moderate, or vigorous) by its energy requirements using values derived from the International Physical Activity Questionnaire (IPAQ) study.^23^

### Ascertainment of vascular risk factors, cardiovascular disease, and mortality outcomes

We considered the following VRFs: hypertension, diabetes, high cholesterol, and smoking; and the following CVDs (incident and prevalent): AF, stroke, ischaemic heart disease (IHD), and MI. Mortality outcomes were ascertained from death register data. We considered all-cause and CVD mortality; the latter was defined as primary cause of death recorded as any CVD (ICD10 Chapter IX I00-I99). Incident CVDs and mortality outcomes were considered as those occurring after CMR imaging. The average follow-up time available for HES and mortality data was 4.2 ± 1.2 (range: 2.5–6.9) years.

For ascertainment of prevalent VRFs and CVDs, we referred to baseline verbal interview, documentation of relevant HES codes, or record in UK Biobank algorithmically defined health outcomes (for MI and stroke). For diabetes and high cholesterol, we also referred to biochemistry data (glycosylated haemoglobin >48 mmol/mol and total cholesterol >7 mmol/L, respectively). The approach to ascertainment of VRFs and CVDs along with a full list of ICD codes used is presented in Supplementary data online, *Table S1*.

### Statistical analysis

Statistical analysis was performed using R version 4.0.3^24^ and RStudio Version 1.3.1093.^25^ We included all UK Biobank participants with quality-controlled CMR data available.

We performed orthogonal principal component analysis (PCA) rotation of the five CMR metrics (LAVi, LAEF, LVM : LVEDV, GLS, and LVGFI), creating uncorrelated CMR variables whilst retaining >90% of their individual variance, as described in previous work.^26–28^ Thus, we removed significant interdependencies between the CMR metrics and were able to include the rotated CMR variables together as exposures in the same model. For inclusion to the PCA, LAV, and LAEF contained 0.03% missing values and GLS contained 3.7% missing values that were imputed with the mean. For comparison of LVGFI and LVEF, we created a separate set of PCA rotated CMR metrics replacing LVGFI with LVEF. The PCA loadings are presented in Supplementary data online, *Tables S2* and *S3*. We estimated the independent association of the PCA rotated CMR metrics with VRFs (hypertension, diabetes, high cholesterol, and smoking) and prevalent CVDs (AF, stroke, IHD, and MI) in multivariable logistic regression models, simultaneously modelling all five CMR metrics and adjusting for confounders (age, sex, ethnicity, deprivation, education, physical activity, and BMI). We used Cox proportional hazards regression for incident CVDs and mortality outcomes (with covariate adjustment as before). In associations with incident CVDs, we excluded participants who had already had the same outcome prior to CMR.

We also present associations with individually entered raw CMR metrics. For associations with prevalent diseases, we used multivariable linear regression, considering individual raw CMR metrics as the model outcome, and VRFs and prevalent CVDs as exposure variables. For incident outcomes, we used Cox proportional hazards regression with raw CMR metrics entered individually as exposure variables. We adjusted for confounders as before. In all models, LAV was log-transformed to remove skew. We corrected for multiple comparisons using a false discovery rate of 0.05 across exposure variables.

## Results

### Population characteristics

We studied 25 896 participants for whom CMR data were available (Supplementary data online, *Figure S1*). The cohort had an average age of 62.9 (±7.5) years old; 52% (*n* = 13 488) were women (*Table 1*). The proportion of participants with hypertension, diabetes, high cholesterol, and smoking was 32.7%, 5.7%, 34.5%, and 3.7%, respectively. There was, overall, less socio-economic deprivation than UK national averages. The proportion of participants with prevalent AF, stroke, IHD, and MI at time of CMR was 1.5%, 1.9%, 6.0%, and 2.4%, respectively (*Table 1*).

**Table 1:** Participant characteristics

	Whole sample	Men	Women
	(*n* = 25 896)	(*n* = 12 408)	(*n* = 13 488)
Age at imaging (years)	62.9 (±7.5)	63.6 (±7.6)	62.2 (±7.4)
Townsend deprivation index	−2.7 (−3.9, −0.7)	−2.7 (−4.0, −0.7)	−2.6 (−3.9, −0.7)
Education			
Left school ≤14 years without qualifications	67 (0.3%)	38 (0.3%)	29 (0.2%)
Left school ≥ 15 years without qualifications	1844 (7.1%)	855 (6.9%)	989 (7.3%)
Secondary school qualification	3485 (13.5%)	1313 (10.6%)	2172 (16.1%)
A levels/AS levels or equivalent	1465 (5.7%)	653 (5.3%)	812 (6.0%)
Other professional qualification	7258 (28.0%)	3666 (29.5%)	3592 (26.6%)
Higher education (e.g. university) degree	11 511 (44.5%)	5755 (46.4%)	5756 (42.7%)
Missing	266 (1.0%)	128 (1.0%)	138 (1.0%)
BMI (kg/m^2^)	25.9 (23.5, 28.9)	26.5 (24.3, 29.1)	25.3 (22.8, 28.6)
Physical activity (summed MET-min/week)	1899 (896, 3573)	1971 (958, 3666)	1838 (834, 3514)
Smoker (current)	960 (3.7%)	540 (4.4%)	420 (3.1%)
Hypertension	8471 (32.7%)	4914 (39.6%)	3557 (26.4%)
High cholesterol	8947 (34.5%)	5117 (41.2%)	3830 (28.4%)
Diabetes	1485 (5.7%)	924 (7.4%)	561 (4.2%)
Prevalent cardiovascular disease			
Atrial fibrillation	386 (1.5%)	273 (2.2%)	113 (0.8%)
Stroke	503 (1.9%)	320 (2.6%)	183 (1.4%)
IHD	1560 (6.0%)	1092 (8.8%)	468 (3.5%)
MI	633 (2.4%)	502 (4.0%)	131 (1.0%)
Incident CVD and mortality outcomes			
Atrial fibrillation	180 (0.7%)	127 (1.0%)	53 (0.4%)
Stroke	178 (0.7%)	114 (0.9%)	64 (0.5%)
IHD	530 (2.0%)	347 (2.8%)	183 (1.4%)
MI	197 (0.8%)	140 (1.1%)	57 (0.4%)
All-cause mortality	331 (1.3%)	220 (1.8%)	111 (0.8%)
CVD mortality	58 (0.2%)	44 (0.4%)	14 (0.1%)
Any of AF, stroke, IHD, MI, or CVD death	880 (3.4%)	583 (4.7%)	297 (2.2%)
CMR metrics			
LAV (mL)	70.0 (57.0, 85.3)	75.9 (61.1, 92.6)	65.9 (54.5, 78.7)
LAVi (mL/m^2^)	38.0 (31.5, 45.4)	38.0 (30.9, 46.0)	38.1 (31.9, 45.0)
LAEF (%)	61.3 (±9.1)	60.6 (±9.6)	61.9 (±8.5)
LVM : LVEDV (g/mL)	0.57 (0.52, 0.63)	0.60 (0.56, 0.66)	0.54 (0.50, 0.59)
LVSVi (mL/m^2^)	47.1 (±8.4)	48.8 (±9.1)	45.6 (±7.4)
LVEF (%)	59.6 (±6.1)	57.8 (±6.1)	61.1 (±5.5)
LVGFI (%)	47.7 (±6.8)	44.8 (±6.2)	50.4 (±6.2)
GLS (%)	−18.5 (±2.7)	−17.8 (±2.6)	−19.1 (±2.7)

Counts variables are presented as number (percentage), continuous variables as mean (standard deviation) or median (inter-quartile range) based on skew.

BMI, body mass index; CMR, cardiovascular magnetic resonance; CVD, cardiovascular disease; GLS, global longitudinal strain; i, indexation to body surface area; IHD, ischaemic heart disease; LAEF, left atrial ejection fraction; LAV, maximum left atrial volume; LVEDV, left ventricular end-diastolic volume; LVEF, left ventricular ejection fraction; LVM, left ventricular mass; LVGFI, left ventricular global function index; MET, metabolic equivalent; MI, myocardial infarction.

**Figure 1:**
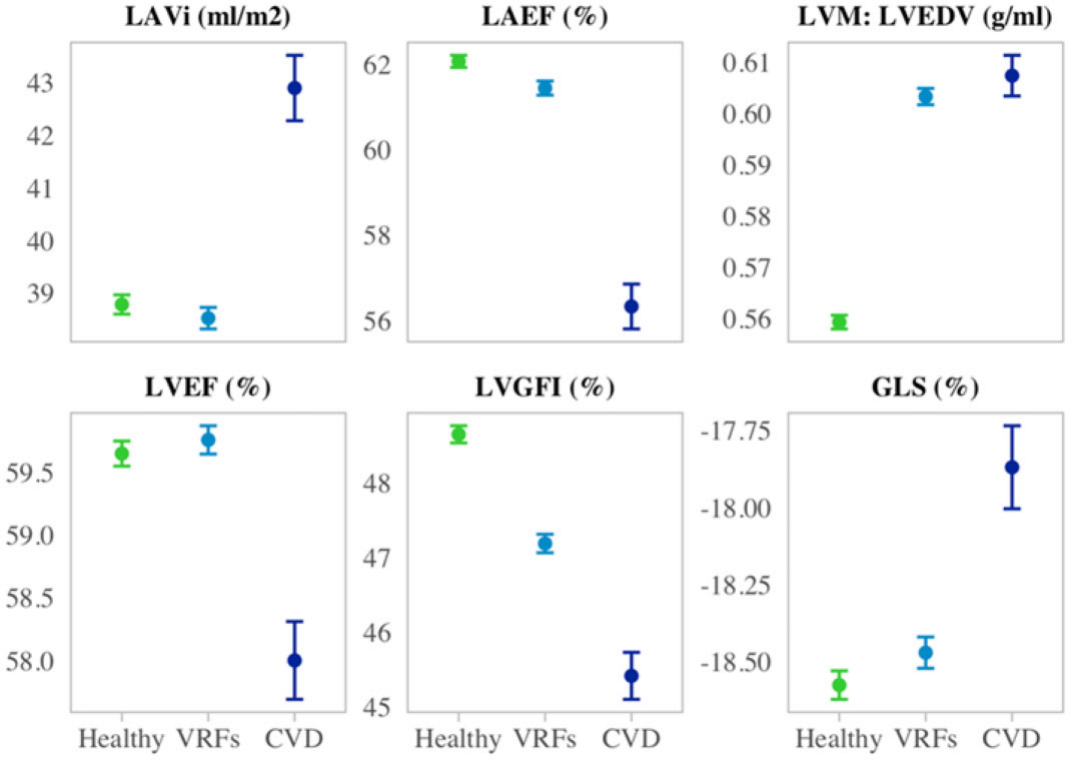
CMR metric means, and 95% confidence interval of the mean stratified by disease status. Within the ‘Healthy’, ‘VRFs’, and ‘CVD’ subsets, we include participants without prevalent CVD or VRFs, with VRFs but without prevalent CVDs, and with prevalent CVDs, respectively. CMR, cardiovascular magnetic resonance; CVD, cardiovascular disease; LVGFI, left ventricular global function index; GLS, global longitudinal strain; i, indexation to body surface area; LAEF, left atrial ejection fraction; LAV, maximum left atrial volume; LVEDV, left ventricular end-diastolic volume; LVEF, left ventricular ejection fraction; left ventricular mass; MI, myocardial infarction.

LA size and function were comparable in men and women after adjustment for body size (*Table 1*). Compared with women, men had, on average, more concentric LV remodelling patterns (higher LVM : LVEDV) and poorer LV function by GLS, LVGFI, and LVEF (*Table 1*). We additionally examined CMR metrics in subsets of participants (i)without VRFs or CVD (healthy), (ii)with VRFs, but without CVD, and (iii)with CVD (*Figure 1* and Supplementary data online, *Table S4*). There was a stepwise decline in LV (by LVGFI and GLS) and LA function (by LAEF) from the healthy subset to those with VRFs and to those with CVD (*Figure 1*). Average LVEF was higher in the participants with VRFs compared with the healthy subset and lower than both subsets in those with CVD. Individuals with VRFs had smaller LAVi than healthy participants; those with CVD had the largest LAVi. The VRF and CVDs subset had higher LVM : LVEDV than the healthy cohort (*Figure 1* and Supplementary data online, *Table S4*).

Over the average follow-up time of 4.2 ± 1.2 years, we observed incidence of 180 (0.7%) AF, 178 (0.7%) stroke, 530 (2.0%) IHD, and 197 (0.8%) MI events. There were 331 deaths during the available follow-up period; of these, 58 were attributed to CVD. In total, 880 (3.4%) participants had at least one incident event, of these 34% (*n* = 297) were women (Supplementary data online, *Table S5*). Participants who experienced an incident event had higher burden of VRFs than the whole cohort, with hypertension, diabetes, high cholesterol, and smoking documented in 50.5%, 9.9%, 46.7%, and 3.9%, respectively (Supplementary data online, *Table S5*).

### Association of CMR metrics with vascular risk factors

In fully adjusted logistic regression models, including all the PCA rotated CMR metrics, we observed association of all the VRFs with poorer LA function (lower LAEF), with statistically significant relationships observed with diabetes and smoking (*Table 2*). Diabetes, high cholesterol, and smoking were associated with smaller LA sizes (lower LAV), whilst hypertension was associated with larger LA size (*Table 2*). There was significant association of all the VRFs with concentric LV remodelling patterns (higher LVM : LVEDV). Hypertension, diabetes, and smoking were associated with significantly poorer LV function by LVGFI and GLS (*Table 2*). In mutually adjusted models with LVEF instead of LVGFI, diabetes was associated with significantly lower LVEF; associations of LVEF with other VRFs were not statistically significant (Supplementary data online, *Table S6*). There was a similar pattern of associations in models using raw CMR metrics entered individually (Supplementary data online, *Table S7*).

**Table 2:** Associations of mutually adjusted CMR metrics with vascular risk factors and prevalent cardiovascular disease in multivariable logistic regression models with full confounder adjustment

	Vascular risk factors				Prevalent cardiovascular disease			
CMR metric	Hypertension	Diabetes	High cholesterol	Smoking (current)	AF	Stroke	IHD	MI
LAVi (mL/m^2^)	1.24^*^	0.87^*^	0.96^*^	0.88^*^	1.30^*^	0.96	1.14^*^	1.09
	(1.21–1.28)	(0.83–0.92)	[0.94, 0.99]	[0.83, 0.94]	[1.18, 1.44]	[0.88, 1.05]	[1.08, 1.20]	[1.01, 1.18]
	7.59 × 10^−46^	1.31 × 10^−6^	0.0129	1.65 × 10^−4^	4.15 × 10^−7^	0.3661	2.73 × 10^−6^	0.0377
LAEF (%)	0.99	0.94^*^	0.99	0.93^*^	0.40^*^	0.88^*^	0.82^*^	0.82^*^
	(0.96–1.02)	(0.89–0.98)	[0.96, 1.02]	[0.87, 0.99]	[0.36, 0.43]	[0.82, 0.96]	[0.78, 0.86]	[0.76, 0.88]
	0.6064	0.0110	0.3950	0.0266	6.15 × 10^−91^	0.0027	1.36 × 10^−14^	3.82 × 10^−8^
LVM : LVEDV	1.43^*^	1.20^*^	1.10^*^	1.29^*^	0.80^*^	1.04	0.85^*^	0.75^*^
	(1.38–1.48)	(1.14–1.27)	[1.07, 1.14]	[1.21, 1.38]	[0.71, 0.90]	[0.95, 1.14]	[0.81, 0.91]	[0.68, 0.82]
	2.40 × 10^−99^	1.10 × 10^−11^	6.28 × 10^−9^	1.05 × 10^−13^	2.16 × 10^−4^	0.4062	1.19 × 10^−7^	1.52 × 10^−10^
LVGFI (%)	0.93^*^	0.87^*^	1.00	0.88^*^	1.23^*^	0.95	0.88^*^	0.71^*^
	(0.90–0.96)	(0.82–0.92)	[0.96, 1.03]	[0.82, 0.94]	[1.11, 1.37]	[0.87, 1.05]	[0.84, 0.94]	[0.65, 0.77]
	1.07 × 10^−5^	1.89 × 10^−6^	0.7519	2.43 × 10^−4^	8.50 × 10^−5^	0.3310	2.63 × 10^−5^	7.96 × 10^−16^
GLS (%)	1.03^*^	1.15^*^	0.97	1.12^*^	1.11	1.04	1.01	1.07
	(1.00–1.07)	(1.09–1.21)	[0.94, 1.00]	[1.05, 1.20]	[1.01, 1.22]	[0.96, 1.14]	[0.95, 1.06]	[0.99, 1.16]
	0.0251	9.34 × 10^−7^	0.0382	9.47 × 10^−4^	0.0387	0.3420	0.8414	0.0836

Results are odds ratios, 95% confidence intervals, and *P*-values. Models are logistic regression models with disease of interest entered as the response (outcome) variable. For the vascular risk factor models, covariates include mutually entered PCA rotated CMR metrics (LAV, LAEF, LVM/LVEDV, GLS, and LVGLFI), age, sex, ethnicity, deprivation, education, body mass index, physical activity, and all the VRFs (except the one set as the model outcome). For the prevalent cardiovascular disease models covariates include mutually entered PCA rotated CMR metrics (LAV, LAEF, LVM/LVEDV, GLS, and LVGLFI), age, sex, ethnicity, deprivation, education, body mass index, physical activity, hypertension, high cholesterol, diabetes, and smoking.

AF, atrial fibrillation; CMR, cardiovascular magnetic resonance; LVGFI, left ventricular global function index; GLS, global longitudinal strain; i, indexation to body surface area; IHD, ischaemic heart disease; LAEF, left atrial ejection fraction; LAV, maximum left atrial volume; LVEDV, left ventricular end-diastolic volume; LVM, left ventricular mass; MI, myocardial infarction; PCA, principal component analysis.

* Statistically significant *P*-values with a false discovery rate of 0.05, giving an approximate threshold of 0.025.

### Association of CMR metrics with prevalent cardiovascular disease

In fully adjusted logistic regression models, including all the PCA rotated CMR metrics, all the prevalent CVDs were associated with significantly lower LAEF (*Table 2*). AF and IHD were associated with significantly larger LA sizes (*Table 2*). As expected, these relationships appeared most dominant for AF (*Table 2*). AF, IHD, and MI were associated with more eccentric LV remodelling pattern (lower LVM : LVEDV). IHD and MI were associated with poorer LV function by LVGFI (*Table 2*). The same pattern of associations was observed with LVEF in mutually adjusted models with LVEF instead of LVGFI (Supplementary data online, *Table S6*). These relationships were broadly similar in models using individual raw CMR metrics; in these models, MI and AF were additionally associated with significantly poorer GLS, LVGFI, and LVEF, but these relationships were attenuated in the mutually adjusted models (Supplementary data online, *Table S7*).

### Association of CMR metrics with incident cardiovascular disease

In fully adjusted Cox regression models, with mutual inclusion of all the PCA rotated CMR metrics, poorer LA function (lower LAEF) was associated with significantly higher risk of incidence of all the CVDs considered, specifically AF, stroke, IHD, and MI (*Table 3* and *Graphical Abstract*). Larger LA size was associated with significantly higher risk of incident AF. More concentric LV remodelling patterns (higher LVM : LVEDV) were associated with significantly increased risk of incident stroke and incident IHD (*Table 3*). Lower LVGFI was associated with significantly higher risk of incident IHD (*Table 3*). In mutually adjusted models with LVEF instead of LVGFI, there was no significant association between LVEF and any of the incident CVDs (Supplementary data online, *Table S8*). In equivalent Cox regression models with raw individually entered CMR metrics, the associations with LA metrics were largely unchanged (Supplementary data online, *Table S9*). In these models, AF, stroke, and IHD were associated with significantly lower LVGFI, stroke, and IHD were associated with significantly poorer GLS, and AF was associated with lower LVEF (Supplementary data online, *Table S9*); these relationships (with exception of IHD and LVGFI) were attenuated in models mutually adjusting for all the CMR metrics (*Table 3*).

**Table 3:** Associations of mutually adjusted CMR metrics with incident cardiovascular disease and mortality outcomes in Cox proportional hazard models with full confounder adjustment

CMR metric	AF	Stroke	IHD	MI	All-cause mortality	CVD mortality
LAVi (mL/m^2^)	1.47^*^	1.13	1.10	1.06	1.11	1.34^*^
	(1.28–1.70)	(0.98–1.31)	(1.01–1.19)	(0.93–1.21)	(1.00–1.23)	(1.05–1.71)
	8.46 × 10^−8^	0.0807	0.0302	0.4002	0.0407	0.0185
LAEF (%)	0.64^*^	0.83^*^	0.88^*^	0.87^*^	0.96	0.85
	(0.56–0.73)	(0.73–0.95)	(0.81–0.95)	(0.76–0.99)	(0.87–1.06)	(0.69–1.04)
	2.50 × 10^−11^	0.0060	9.95 × 10^−4^	0.0294	0.4029	0.1119
LVM : LVEDV	1.06	1.22^*^	1.27^*^	1.14	1.09	1.05
	(0.92–1.23)	(1.06–1.40)	(1.17–1.37)	(0.99–1.30)	(0.98–1.22)	(0.82–1.35)
	0.4036	0.0065	1.27x10^−8^	0.0732	0.0959	0.7039
LVGFI (%)	0.92	0.89	0.88^*^	0.95	0.85^*^	0.61^*^
	(0.79–1.06)	(0.76–1.03)	(0.80–0.96)	(0.82–1.11)	(0.76–0.95)	(0.48–0.78)
	0.2521	0.1266	0.0063	0.5372	0.0050	5.95 × 10^−5^
GLS (%)	0.97	1.10	1.08	1.05	1.14^*^	1.10
	(0.85–1.12)	(0.95–1.27)	(0.99–1.17)	(0.91–1.20)	(1.02–1.27)	(0.87–1.38)
	0.7171	0.1880	0.0888	0.5254	0.0170	0.4314

Results are hazard ratios, 95% confidence intervals, and *P*-values. Covariates are LAV, LAEF, LVM/LVEDV, GLS, GLFI, age, sex, ethnicity, deprivation, education, body mass index, hypertension, high cholesterol, diabetes, physical activity, and smoking. The CMR variables are principal component analysis rotated variables.

AF, atrial fibrillation; CMR, cardiovascular magnetic resonance; CVD, cardiovascular disease; LVGFI, left ventricular global function index; GLS, global longitudinal strain; i, indicates indexation to body surface area; IHD, ischaemic heart disease; LAEF, left atrial ejection fraction; LAV, maximum left atrial volume; LVEDV, left ventricular end-diastolic volume; LVM, left ventricular mass; MI, myocardial infarction.

* Statistically significant *P*-values with a false discovery rate of 0.05, giving an approximate threshold of 0.028.

### Association of CMR metrics with mortality outcomes

In fully adjusted Cox regression models, including all the PCA rotated CMR metrics, larger LAVi was associated with significantly greater hazard of CVD mortality. Poorer GLS was associated with significantly higher risk of all-cause mortality. Lower LVGFI was associated with significantly higher risk of both all-cause and CVD mortality (*Table 3*). In mutually adjusted models with LVEF instead of LVGFI, LVEF was also associated with significantly lower risk of all-cause and CVD mortality, but with slightly smaller effect sizes than LVGFI (Supplementary data online, *Table S8* and *Table 3*).

## Discussion

### Summary of findings

In this study of 25 896 UK Biobank participants, we demonstrate associations of CMR derived LA structure and function metrics with VRFs, prevalent CVDs, incident CVDs, and mortality outcomes, independent of LV measures and a wide range of clinical confounders.

Lower LAEF emerged as a consistent and independent indicator of VRFs (diabetes and smoking) and prevalent and incident CVDs (AF, stroke, IHD, and MI). Diabetes, high cholesterol, and smoking were associated with smaller LAV. Hypertension and IHD were associated with larger LAV, perhaps reflecting more advanced diastolic dysfunction in these conditions. Both prevalent and incident AF were associated with larger LA sizes. More concentric LV remodelling patterns were associated with VRFs and incident CVDs, whilst prevalent CVDs were associated with more eccentric LV remodelling. These observations likely reflect differential dominance of LV pressure and volume overload in the transition from risk factor to disease, with volume overload becoming dominant after disease occurrence. LVGFI, GLS, and LVEF provided good indications of VRFs and prevalent CVDs, with LVGFI showing the most consistent results. LVGFI and LVEF were independent predictors of all-cause and CVD mortality, with larger effect sizes observed with LVGFI. Higher LAVi was independently associated with significantly higher CVD mortality.

Both the LV and LA metrics were independently informative in associations with risk factors and prevalent disease. In associations with incident outcomes many of the LV associations were attenuated, whilst LAEF associations with all incident CVDs remained robust independent of LV metrics and other confounders. Larger LAVi appeared a strong independent predictor for CVD mortality. These observations demonstrate the independent utility of LA structure and function metrics, particularly for prediction of incident outcomes, which likely reflects pre-clinical LA remodelling before establishment of LV alterations.

### Comparison with existing research

We observed strong and significant associations of lower LAEF and larger LAV with both prevalent and incident AF. Consistently, Bertelsen *et al.*^29^ also demonstrate significant association of larger CMR-derived LA volumes and poorer LA function with greater risk of AF detected on an implantable loop recorder in 203 participants with stroke risk factors but without pre-existing AF.^29^ These LA alterations likely reflect underlying atrial remodelling, which predisposes to (and can also occur as a result of) AF. In a study of 1148 MESA (Multi-Ethnic Study of Atherosclerosis) participants, Heckbert *et al.*^30^ demonstrate association of lower total LAEF and larger LAV with greater burden of premature atrial contractions on ambulatory electrocardiographic monitoring; such arrhythmias may be precursors of AF and indicative of atrial fibrosis. Indeed, in a study of 111 patients without a prior history of atrial arrhythmia, Quail *et al.*^11^ demonstrate association of LA late gadolinium enhancement (a marker of atrial fibrosis) with incident atrial arrhythmias.

We observed association of poorer LAEF with both prevalent and incident stroke independent of other CMR metrics. Larger LAV was associated with significantly greater risk of incident stroke in individual models, but not in models including other CMR metrics. In a study of 169 patients with AF referred for catheter ablation, Inoue *et al.*^12^ similarly demonstrate the association of poorer LA function (LAEF) with prior stroke or transient ischaemic attack. Habibi *et al.*^31^ also report significant association of lower LAEF, but not LA size, with incident ischaemic stroke in 4261 MESA participants. We additionally demonstrate significant associations of lower LAEF with incident IHD and incident MI, independent of other CMR metrics. In a study of 536 diabetic MESA participants without clinical CVD, Markman *et al.*^32^ also demonstrate the association of poorer LA function with incident CVD (defined as composite of MI, resuscitated cardiac arrest, angina, stroke, heart failure, or AF). Our findings add to the literature by demonstrating specific independent association of larger LAVi and higher CVD mortality risk.

We observed association of diabetes with smaller LAV and lower LAEF, along with more concentric LV remodelling and poorer LV function metrics. Two studies of small diabetic cohorts have also demonstrated lower LAEF in diabetics compared with controls but have not demonstrated any significant difference in LA size.^10,^^33^ Similar to our observations, studies using the first release of the UK Biobank CMR data have demonstrated association of diabetes with smaller atrial volumes.^34,^^35^ Conversely, some echocardiography studies have demonstrated association of diabetes with larger LA sizes. For example, Armstrong *et al.*^9^ demonstrate association of larger LA diameter with prevalent diabetes in 2903 CARDIA study participants. LA alterations evolve with disease progression, with LA dilatation reflecting persistently elevated LV filling pressures and advancement of diastolic (and systolic) LV dysfunction.^3^ Thus, the duration of exposure to and control of the diabetes, as well as the overall risk factor profile of participants likely influence associations with LA size. As the UK Biobank comprises a relatively healthy cohort, our observations reflect milder disease. Indeed, we observed significant association of larger LAV with pre-existing IHD, a condition associated with more advanced LV impairment. This is further supported by the observed association of larger LAV with greater CVD mortality risk. In our study, and in existing literature, LAEF appears as a reliable and consistent indicator of diabetes and other key morbidities.

We observed the association of more concentric LV remodelling patterns with VRFs and incident CVDs, whilst prevalent CVDs were associated with more eccentric LV remodelling patterns. These observations likely reflect predominance of pressure overload in the presence of VRFs and prior to disease occurrence, but dominance of volume overload after development of clinical CVD. Our analysis also demonstrates consistent and significant association of lower LVGFI with VRFs, prevalent CVDs, incident IHD, and higher risk of all-cause and CVD mortality. In separate analyses comparing LVGFI to LVEF, the latter showed similar but less consistent associations. Our findings add strength to existing studies which have proposed the high utility of this LVGFI as a measure of LV function.^20,^^21^

### Clinical implications

In this study of 25 896 UK Biobank participants, we describe independent clinical associations of CMR derived measures of LA structure and function (LAV and LAEF). These metrics, particularly LAEF, show robust associations with key cardiovascular outcomes independent of LV measures. Thus, there is potential utility for these metrics as components of clinical risk prediction algorithms. In the next stages towards development of such clinical models, there is need for evaluation of clinical relationships in other cohorts and settings. Any proposed clinical risk stratification models will require careful validation and evaluation of model performance prior to use in clinical practice.

### Strengths and limitations

The highly detailed participant characterization and standardized CMR data in the UK Biobank permitted evaluation of associations of CMR phenotypes with key VRFs and CVDs in a very large cohort, whilst considering a wide range of confounders. The linked reliably recorded health outcome data also permitted assessment of associations with incident CVDs. The duration of follow up was relatively short and the proportion of participants with incident events was small (*n* = 880/25 896, 3.4%), and even fewer when considering subgroups of participants (Supplementary data online, *Table S10*). Given that this limits our power to detect statistically significant associations with incident events, the observed significant relationships between LA metrics and incident CVDs are all the more notable. Identification of incident outcomes using HES is ideal for conditions such as acute MI and stroke, which almost always require hospitalization. However, this approach is not optimal for endpoints that do not always require hospital admission, such as diastolic heart failure or mitral valve disease, which we were unable to consider in the analysis. There were few CVD mortality events, which means that analysis with this outcome is likely underpowered to appreciate the full picture of CMR associations (particularly small and moderate effect sizes). As events accrue in the UK Biobank, more adequately powered analyses may be conducted with possibility of evaluating associations with more granular disease-specific mortality outcomes. Finally, due to the observational nature of the study, we cannot exclude residual confounding or reverse causation; however, the primary aim of the present study is description of associations rather than causal inference.

## Conclusions

LA structure and function measures (LAEF and LAV) demonstrate significant associations with key prevalent and incident cardiovascular outcomes, independent of LV metrics. These measures have potential clinical utility for disease discrimination and outcome prediction.

## Supplementary data

Supplementary data are available at *European Heart Journal - Cardiovascular Imaging* online.

## Supplementary Material

jeab266_Supplementary_Data

## Data Availability

The data underlying this article were provided by the UK Biobank under access application 2964. UK Biobank will make the data available to bona fide researchers for all types of health-related research that is in the public interest, without preferential or exclusive access for any persons. All researchers will be subject to the same application process and approval criteria as specified by UK Biobank. For more details on the access procedure, see the UK Biobank website: http://www.ukbiobank.ac.uk/register-apply/.
