# Corrigendum to: Left atrial structure and function are associated with cardiovascular outcomes independent of left ventricular measures: a UK Biobank CMR study

**DOI:** 10.1093/ehjci/jeac119

**Published:** 2022-06-15

**Authors:** Zahra Raisi-Estabragh, Celeste McCracken, Dorina Gabriela Condurache, Nay Aung, Jose D Vargas, Hafiz Naderi, Patricia B Munroe, Stefan Neubauer, Nicholas C Harvey, Steffen E Petersen

*European Heart Journal - Cardiovascular Imaging*, jeab266, https://doi.org/10.1093/ehjci/jeab266

In the originally published version of this manuscript, there was an error in the third author’s name. The third author’s name should read “Dorina-Gabriela Condurache” instead of “Dorina Condurache”. This error has been corrected.

