# Supplementary material for: Left atrial structure and function are associated with cardiovascular outcomes independent of left ventricular measures: a UK Biobank CMR study
Source: Eur Heart J Cardiovasc Imaging. Author manuscript; Available in PMC 2022 Aug 22. (PMC9365306; doi:10.1093/ehjci/jeab266)
Supplement: Supplementary Figure 1 [file EMS151232-supplement-Supplementary_Figure_1.docx]

**Supplementary Figure 1. Flow chart of participant selection**


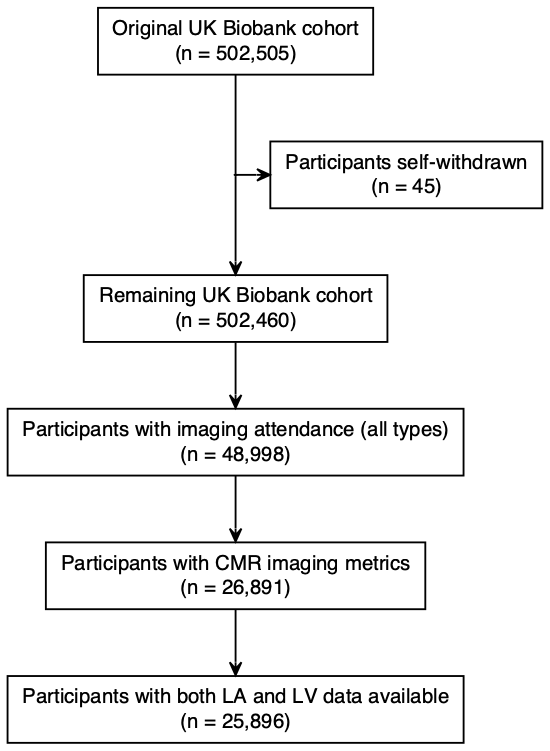


**Supplementary Figure 1 footnote.** CMR: cardiovascular magnetic resonance, LA: left atrium, LV: left ventricle.
