# Supplementary material for: Left atrial structure and function are associated with cardiovascular outcomes independent of left ventricular measures: a UK Biobank CMR study
Source: Eur Heart J Cardiovasc Imaging. Author manuscript; Available in PMC 2022 Aug 22. (PMC9365306; doi:10.1093/ehjci/jeab266)
Supplement: Supplementary Table 1-10 [file EMS151232-supplement-Supplementary_Table_1_10.docx]

**Supplementary Table 1. Approach to ascertainment of cardiovascular risk factors and diseases**

| **Source** | **field ID or ICD code** | **Description** |
| --- | --- | --- |
| Ischaemic heart disease |  |  |
| Self-report (Field ID 20002) |  | angina |
| Self-report (Field ID 20002) |  | heart attack/myocardial infarction* |
| Algorithm | 42000 | 42000 Date of myocardial infarction* |
| ICD10 | I20 | I20 Angina pectoris |
| ICD10 | I21 | I21 Acute myocardial infarction* |
| ICD10 | I22 | I22 Subsequent myocardial infarction* |
| ICD10 | I23 | I23 Certain current complications following acute myocardial infarction |
| ICD10 | I24 | I24 Other acute ischaemic heart diseases |
| ICD10 | I25 | I25 Chronic ischaemic heart disease |
| First occurrences | 131296 | angina pectoris |
| First occurrences | 131298 | acute myocardial infarction* |
| First occurrences | 131300 | subsequent myocardial infarction* |
| First occurrences | 131302 | certain current complications following acute myocardial infarction |
| First occurrences | 131304 | other acute ischaemic heart diseases |
| First occurrences | 131306 | chronic ischaemic heart disease |
| Diagnosed by doctor (Field ID 6150) | 6150: 2 | Angina |
| Diagnosed by doctor | 3627 | Age angina diagnosed |
| Diagnosed by doctor (Field ID 6150) | 6150: 1 | Heart attack* |
| Diagnosed by doctor | 3894 | Age heart attack diagnosed* |
| ICD9 | 410 | 410 Acute myocardial infarction* |
| ICD9 | 411 | 411 Other acute and subacute forms of ischaemic heart disease |
| ICD9 | 412 | 412 Old myocardial infarction* |
| Atrial fibrillation |  |  |
| Self-report (Field ID 20002) |  | atrial fibrillation |
| ICD10 | I480 | I48.0 Paroxysmal atrial fibrillation |
| ICD10 | I481 | I48.1 Persistent atrial fibrillation |
| ICD10 | I482 | I48.2 Chronic atrial fibrillation |
| Stroke |  |  |
| Self-report (Field ID 20002) | stroke | stroke |
| Algorithm | 42006 | 42006 Date of stroke |
| ICD10 | I64 | I64 Stroke, not specified as haemorrhage or infarction |
| Diagnosed by doctor (Field ID 6150) | 6150: 3 | Stroke |
| Diagnosed by doctor | 4056 | Age stroke diagnosed |
| First occurrences | 131368 | Date I64 first reported (stroke, not specified as haemorrhage or infarction) |
| Self-report (Field ID 20002) |  | ischaemic stroke |
| Algorithm | 42008 | 42008 Date of ischaemic stroke |
| ICD10 | I63 | I63 Cerebral infarction |
| First occurrences | 131366 | cerebral infarction |
| Self-report (Field ID 20002) |  | brain haemorrhage |
| Algorithm | 42010 | 42010 Date of intracerebral haemorrhage (should be covered by 42006) |
| ICD10 | I61 | I61 Intracerebral haemorrhage |
| ICD9 | 431 | 431 Intracerebral haemorrhage |
| ICD10 | I62 | I62 Other nontraumatic intracranial haemorrhage |
| First occurrences | 131362 | intracerebral haemorrhage |
| First occurrences | 131364 | other nontraumatic intracranial haemorrhage |
| ICD9 | 432 | 432 Other and unspecified intracranial haemorrhage |
| Diabetes |  |  |
| Diagnosed by doctor | 2443 | Diabetes diagnosed by doctor |
| Diagnosed by doctor | [2976](https://biobank.ndph.ox.ac.uk/showcase/field.cgi?id=2976) | Age diabetes diagnosed by doctor |
| Self-report (Field ID 20002) |  | diabetes |
| Self-report (Field ID 20002) |  | type 1 diabetes |
| Self-report (Field ID 20002) |  | type 2 diabetes |
| ICD10 | E10 | Type 1 diabetes mellitus |
| ICD10 | E11 | Type 2 diabetes mellitus |
| ICD10 | E13 | Other specified diabetes mellitus |
| ICD10 | E14 | Unspecified diabetes mellitus |
| ICD10 | G590 | Diabetic mononeuropathy |
| ICD10 | G632 | Diabetic polyneuropathy |
| ICD10 | H280 | Diabetic cataract |
| ICD10 | H360 | Diabetic retinopathy |
| ICD10 | M142 | Diabetic arthropathy |
| ICD10 | N083 | Glomerular disorders in diabetes mellitus |
| ICD10 | O240 | Diabetes mellitus in pregnancy: Pre-existing type 1 diabetes mellitus |
| ICD10 | O241 | Diabetes mellitus in pregnancy: Pre-existing type 2 diabetes mellitus |
| ICD10 | O243 | Diabetes mellitus in pregnancy: Pre-existing diabetes mellitus, unspecified |
| ICD10 | O244 | Diabetes mellitus arising in pregnancy |
| ICD10 | O249 | Diabetes mellitus in pregnancy, unspecified |
| ICD10 | Y423 | Insulin and oral hypoglycaemic [antidiabetic] drugs |
| First occurrences | 130706 | Date E10 first reported (insulin-dependent diabetes mellitus) |
| First occurrences | 130708 | Date E11 first reported (non-insulin-dependent diabetes mellitus) |
| First occurrences | 130712 | Date E13 first reported (other specified diabetes mellitus) |
| First occurrences | 130714 | Date E14 first reported (unspecified diabetes mellitus) |
| Medications (6177, 6153) | 3 | Insulin |
| Biochemistry | 30750 | Glycated haemoglobin (HbA1c) |
| ICD9 | 250 | Diabetes mellitus |
| High cholesterol |  |  |
| ICD10 | E780 | Pure hypercholesterolaemia |
| ICD10 | E782 | Mixed hyperlipidaemia |
| ICD10 | E783 | Hyperchylomicronaemia |
| ICD10 | E784 | Other hyperlipidaemia |
| ICD10 | E785 | Hyperlipidaemia, unspecified |
| First occurrences | 130814 | Date E78 first reported (disorders of lipoprotein metabolism and other lipidaemias) |
| Medications (6177, 6153) | 1 | Cholesterol lowering medication |
| Biochemistry | 30690 | Cholesterol |
| Hypertension |  |  |
| Self-report (Field ID 20002) |  | essential hypertension |
| Self-report (Field ID 20002) |  | hypertension |
| ICD10 | I10 | Essential (primary) hypertension |
| Medications (6177, 6153) | 2 | Blood pressure medication |
| First occurrences | 131286 | Date I10 first reported (essential (primary) hypertension) |
| Diagnosed by doctor (Field ID 6150) | 6150: 4 | High blood pressure |
| Diagnosed by doctor | 2966 | Age high blood pressure diagnosed |

**Supplementary Table 1 footnote.** ICD: international classification of disease.

**Supplementary Table 2. CMR variable component loadings from Principal component analysis (includes LVGFI)**

| CMR Metric | Rotated LVGFI | Rotated LAVi | Rotated GLS | Rotated LAEF | Rotated LVM: LVEDV |
| --- | --- | --- | --- | --- | --- |
| LV GFI (%)% | **0.926** | 0.049 | -0.249 | 0.117 | -0.252 |
| LAVi (log) (ml/m^2^) | 0.048 | **0.956** | -0.031 | -0.26 | -0.125 |
| GLS (%) | -0.225 | -0.028 | **0.961** | -0.137 | 0.083 |
| LAEF (%) | 0.114 | -0.272 | -0.147 | **0.943** | -0.048 |
| LVM: LVEDV (g/ml) | -0.226 | -0.121 | 0.083 | -0.042 | **0.962** |

**Supplementary Table 2 footnote.** CMR: cardiovascular magnetic resonance, LAEF: left atrial ejection fraction, LAV: maximum left atrial volume, LVEDV: left ventricular end-diastolic volume, LVM: left ventricular mass, LVGFI: LV global function index.

**Supplementary Table 3. CMR variable component loadings from Principal component analysis (includes LVEF)**

| CMR Metric | Rotated LVEF | Rotated LAVi | Rotated LVM: LVEDV | Rotated GLS | Rotated LAEF |
| --- | --- | --- | --- | --- | --- |
| LVEF (%) | **0.969** | 0.005 | -0.013 | -0.221 | 0.109 |
| LAVi (log) (ml/m^2^) | 0.007 | **0.956** | -0.135 | -0.033 | -0.259 |
| LVM: LVEDV (g/ml) | -0.013 | -0.121 | **0.986** | 0.101 | -0.051 |
| GLS (%) | -0.23 | -0.03 | 0.11 | **0.957** | -0.136 |
| LAEF (%) | 0.119 | -0.271 | -0.061 | -0.144 | **0.942** |

**Supplementary Table 3 footnote.** CMR: cardiovascular magnetic resonance, LAEF: left atrial ejection fraction, LAV: maximum left atrial volume, LVEDV: left ventricular end-diastolic volume, LVM: left ventricular mass, LVEF: left ventricular ejection fraction.

**Supplementary Table 4. Summary of CMR metric means and 95% confidence interval of the mean stratified by disease status with confounder adjusted between group comparisons**

| CMR Metric | Without CVD or VRFs | With VRF(s), but without CVD | Only those with CVD | p-value ^c^ |
| --- | --- | --- | --- | --- |
| Sample size (n) | 12,393 | 11,237 | 2,266 | – |
| LAVi (ml/m^2^) | 38.8 | 38.5 | 42.9 ^a,b^ | < 0.001 |
|  | [38.6, 39.0] | [38.3, 38.7] | [42.3, 43.5] |  |
| LAEF (%) | 62.1 | 61.4 | 56.3 ^a,b^ | < 0.001 |
|  | [61.9, 62.2] | [61.3, 61.6] | [55.8, 56.9] |  |
| LVM: LVEDV (g/ml) | 0.559 | 0.603 ^a^ | 0.607 ^a^ | < 0.001 |
|  | [0.558, 0.561] | [0.602, 0.605] | [0.603, 0.611] |  |
| LVEF (%) | 59.6 | 59.8 ^a^ | 58 ^a,b^ | < 0.001 |
|  | [59.5, 59.7] | [59.6, 59.9] | [57.7, 58.3] |  |
| LVGFI (%) | 48.6 | 47.2 ^a^ | 45.4 ^a,b^ | < 0.001 |
|  | [48.5, 48.8] | [47.1, 47.3] | [45.1, 45.7] |  |
| GLS (%) | -18.6 | -18.5 ^a^ | -17.9 ^a,b^ | < 0.001 |
|  | [-18.6, -18.5] | [-18.5, -18.4] | [-18.0, -17.7] |  |

**Supplementary Table 4 footnotes.** Without CVD or VRFs: participants without prevalent CVD or VRFs; With VRF(s), but without CVD: participants with at least 1 VRF, but without prevalent CVD; Only those with CVD: only individuals with prevalent CVD. Adjusted models contain age, sex, ethnicity, BMI, Townsend deprivation score, physical activity and education as covariates. ^a^ Mean is significantly different from group without CVD or risk factors with a p-value < 0.015 in an adjusted model. ^b^ Mean is significantly different from group with risk factors with a p-value < 0.015 in an adjusted model. ^c^ Overall p-value for differences between groups in a one-way analysis of variance.

**Supplementary Table 5. Characteristics of participants with at least one incident cardiovascular disease or mortality outcome**

|  | **Whole sample** | **Men** | **Women** |
| --- | --- | --- | --- |
|  | **(n= 880)** | **(n= 583)** | **(n= 297)** |
| Age at imaging (years) | 66.2 (±7.0) | 66.3 (±7.1) | 66.0 (±6.7) |
| Education |  |  |  |
| Left school ≤14yrs without qualifications | 3 (0.3%) | 2 (0.3%) | 1 (0.3%) |
| Left school ≥ 15yrs without qualifications | 79 (9.0%) | 53 (9.1%) | 26 (8.8%) |
| Secondary school completion qualification | 136 (15.5%) | 69 (11.8%) | 67 (22.6%) |
| A levels / AS levels or equivalent | 46 (5.2%) | 34 (5.8%) | 12 (4.0%) |
| Other professional qualification | 266 (30.2%) | 181 (31.0%) | 85 (28.6%) |
| Higher education (e.g., university) degree | 342 (38.9%) | 238 (40.8%) | 104 (35.0%) |
| Missing | 8 (0.9%) | 6 (1.0%) | 2 (0.7%) |
| BMI (kg/m^2^) | 27.2 (±4.1) | 27.5 (±3.8) | 26.7 (±4.6) |
| Physical activity (summed MET-minutes/week) | 1611 [773, 3493] | 1708 [814, 3546] | 1512 [579, 3330] |
| Smoker (current) | 34 (3.9%) | 25 (4.3%) | 9 (3.0%) |
| Hypertension | 444 (50.5%) | 305 (52.3%) | 139 (46.8%) |
| High cholesterol | 411 (46.7%) | 287 (49.2%) | 124 (41.8%) |
| Diabetes | 87 (9.9%) | 67 (11.5%) | 20 (6.7%) |
| CMR Metrics |  |  |  |
| LAV (ml) | 75.5 [60.0, 95.8] | 78.9 [62.2, 98.7] | 69.3 [56.7, 83.4] |
| LAVi (ml/m^2^) | 39.7 [31.9, 48.9] | 39.6 [31.3, 49.3] | 40.0 [33.2, 48.1] |
| LAEF (%) | 57.8 (±11.2) | 57.1 (±11.5) | 59.0 (±10.6) |
| LVM: LVEDV (g/ml) | 0.62 (±0.10) | 0.64 (±0.10) | 0.57 (±0.08) |
| LVSVi (ml/m^2^) | 47.3 (±9.3) | 48.1 (±9.5) | 45.6 (±8.6) |
| LVEF (%) | 59.2 [54.6, 63.4] | 57.9 [53.9, 62.1] | 61.2 [56.2, 64.6] |
| LVGFI (%) | 45.4 (±7.3) | 43.6 (±6.8) | 48.8 (±6.9) |
| GLS (%) | -17.8 (±3.1) | -17.2 (±2.9) | -18.9 (±3.3) |

**Supplementary Table 5 footnote.** CMR: cardiovascular magnetic resonance, LAEF: left atrial ejection fraction, LAV: maximum left atrial volume, LVEDV: left ventricular end-diastolic volume, LVEF: left ventricular ejection fraction, LVM: left ventricular mass, LVGFI: left ventricular global function index, MET: metabolic equivalent.

**Supplementary Table 6. Associations of mutually adjusted CMR metrics with vascular risk factors and prevalent cardiovascular disease in multivariable logistic regression models with full confounder adjustment**

|  | **Vascular risk factors** | | | | **Prevalent cardiovascular disease** | | | |
| --- | --- | --- | --- | --- | --- | --- | --- | --- |
| **CMR Metric** | **Hypertension** | **Diabetes** | **High cholesterol** | **Smoking (current)** | **AF** | **Stroke** | **IHD** | **MI** |
| LAVi (ml/m^2^) | 1.24* | 0.87* | 0.96* | 0.88* | 1.31* | 0.96 | 1.14* | 1.09 |
|  | [1.21, 1.28] | [0.83, 0.92] | [0.94, 0.99] | [0.83, 0.94] | [1.18, 1.45] | [0.88, 1.05] | [1.08, 1.20] | [1.00, 1.18] |
|  | 7.15x10^-46^ | 1.13x10^-6^ | 0.0129 | 1.50x10^-4^ | 3.46x10^-7^ | 0.3648 | 3.21x10^-6^ | 0.0418 |
| LAEF (%) | 0.99 | 0.94* | 0.99 | 0.93* | 0.40* | 0.88* | 0.82* | 0.83* |
|  | [0.96, 1.02] | [0.89, 0.98] | [0.96, 1.01] | [0.87, 0.99] | [0.36, 0.43] | [0.82, 0.96] | [0.78, 0.87] | [0.77, 0.89] |
|  | 0.4488 | 0.0107 | 0.3422 | 0.0234 | 6.14x10^-90^ | 0.0029 | 5.14x10^-14^ | 1.68x10^-7^ |
| LVM: LVEDV | 1.44* | 1.23* | 1.10* | 1.32* | 0.77* | 1.05 | 0.88* | 0.79* |
|  | [1.39, 1.48] | [1.16, 1.30] | [1.06, 1.14] | [1.23, 1.41] | [0.68, 0.87] | [0.95, 1.15] | [0.82, 0.93] | [0.72, 0.86] |
|  | 2.89x10^-97^ | 3.08x10^-13^ | 2.24x10^-8^ | 1.01x10^-14^ | 2.51x10^-5^ | 0.3378 | 1.31x10^-5^ | 3.78x10^-7^ |
| LVEF (%) | 1.01 | 0.90* | 1.02 | 0.93 | 1.13* | 0.96 | 0.86* | 0.68* |
|  | [0.98, 1.04] | [0.86, 0.95] | [0.99, 1.05] | [0.87, 0.99] | [1.02, 1.24] | [0.88, 1.04] | [0.81, 0.90] | [0.63, 0.74] |
|  | 0.6117 | 2.24x10^-4^ | 0.1685 | 0.0311 | 0.0155 | 0.3284 | 5.23x10^-9^ | 4.12x10^-24^ |
| GLS (%) | 1.04* | 1.15* | 0.97 | 1.12* | 1.11 | 1.04 | 1.00 | 1.05 |
|  | [1.01, 1.07] | [1.09, 1.21] | [0.94, 1.00] | [1.05, 1.20] | [1.00, 1.22] | [0.95, 1.14] | [0.94, 1.05] | [0.97, 1.14] |
|  | 0.0060 | 1.05x10^-6^ | 0.0610 | 6.92x10^-4^ | 0.0461 | 0.3612 | 0.8922 | 0.2160 |

**Supplementary Table 6 footnote.** Results are odds ratios, 95% confidence intervals, and p-values. Models are logistic regression models with disease of interest entered as the response (outcome) variable. For the vascular risk factor models, covariates include mutually entered PCA rotated CMR metrics (LAV, LAEF, LVM/LVEDV, GLS, LVEF), age, sex, ethnicity, deprivation, education, body mass index, physical activity, and all the VRFs (except the one set as the model outcome). For the prevalent cardiovascular disease models covariates include mutually entered PCA rotated CMR metrics (LAV, LAEF, LVM/LVEDV, GLS, LVEF), age, sex, ethnicity, deprivation, education, body mass index, physical activity, hypertension, high cholesterol, diabetes, and smoking. *indicates statistically significant p-values with a false discovery rate of 0.05, giving an approximate threshold of 0.025 for this table. AF: atrial fibrillation, CMR: cardiovascular magnetic resonance, LVGFI: left ventricular global function index, GLS: global longitudinal strain, i: indicates indexation to body surface area, IHD: ischaemic heart disease, LAEF: left atrial ejection fraction, LAV: maximum left atrial volume, LVEDV: left ventricular end-diastolic volume, LVM: left ventricular mass, MI: myocardial infarction, PCA: principal component analysis.

**Supplementary Table 7. Associations of vascular risk factors and prevalent cardiovascular disease with individually entered raw CMR metrics in multivariable linear regression models with full confounder adjustment**

|  | **Vascular risk factors** | | | | **Prevalent cardiovascular disease** | | | |
| --- | --- | --- | --- | --- | --- | --- | --- | --- |
| **CMR Metric** | **Hypertension** | **Diabetes** | **High cholesterol** | **Smoker (current)** | **AF** | **Stroke** | **IHD** | **MI** |
| LAVi (ml/m^2^) | 0.16* | -0.13* | -0.04* | -0.15* | 0.86* | -0.00 | 0.24* | 0.07 |
|  | [0.13, 0.19] | [-0.19, -0.08] | [-0.07, -0.01] | [-0.22, -0.09] | [0.77, 0.96] | [-0.09, 0.08] | [0.17, 0.30] | [-0.03, 0.17] |
|  | 3.14x10^-27^ | 1.79x10^-6^ | 0.0086 | 3.22x10^-6^ | 1.08x10^-64^ | 0.9445 | 3.70x10^-12^ | 0.1749 |
| LAEF (%) | -0.09* | -0.11* | -0.00 | -0.07 | -1.51* | -0.13* | -0.23* | -0.22* |
|  | [-0.11, -0.06] | [-0.16, -0.06] | [-0.03, 0.02] | [-0.13, -0.00] | [-1.60, -1.41] | [-0.22, -0.04] | [-0.29, -0.16] | [-0.32, -0.13] |
|  | 2.10x10^-9^ | 5.52x10^-5^ | 0.7751 | 0.0460 | 1.79x10^-196^ | 0.0033 | 1.22x10^-11^ | 6.63x10^-6^ |
| LVM/LVEDV | 0.24* | 0.23* | 0.07* | 0.25* | -0.24* | 0.04 | -0.09* | -0.22* |
| (g/ml) | [0.22, 0.27] | [0.18, 0.27] | [0.05, 0.10] | [0.20, 0.31] | [-0.32, -0.15] | [-0.03, 0.12] | [-0.15, -0.04] | [-0.30, -0.13] |
|  | 1.08x10^-83^ | 1.71x10^-21^ | 2.99x10^-9^ | 6.20x10^-19^ | 1.12x10^-7^ | 0.2646 | 0.0016 | 1.02x10^-6^ |
| LVEF (%) | 0.03* | -0.13* | 0.03 | -0.08* | -0.35* | -0.08 | 0.00 | -0.60* |
|  | [0.01, 0.06] | [-0.18, -0.08] | [-0.00, 0.05] | [-0.14, -0.01] | [-0.45, -0.26] | [-0.16, 0.01] | [-0.06, 0.07] | [-0.70, -0.50] |
|  | 0.0188 | 1.84x10^-6^ | 0.0694 | 0.0161 | 7.57x10^-13^ | 0.0840 | 0.8972 | 4.34x10^-34^ |
| LVGFI (%) | -0.08* | -0.19* | -0.01 | -0.17* | -0.18* | -0.08 | 0.05 | -0.42* |
|  | [-0.10, -0.05] | [-0.24, -0.14] | [-0.04, 0.01] | [-0.23, -0.11] | [-0.27, -0.08] | [-0.16, 0.00] | [-0.01, 0.11] | [-0.51, -0.33] |
|  | 5.99x10^-9^ | 1.66x10^-14^ | 0.3526 | 7.84x10^-9^ | 1.71x10^-4^ | 0.0660 | 0.1080 | 2.98x10^-19^ |
| GLS (%) | 0.05* | 0.22* | -0.02 | 0.16* | 0.52* | 0.08 | -0.04 | 0.29* |
|  | [0.02, 0.08] | [0.17, 0.28] | [-0.05, 0.01] | [0.09, 0.22] | [0.42, 0.62] | [-0.01, 0.16] | [-0.11, 0.02] | [0.19, 0.38] |
|  | 3.02x10^-4^ | 2.27x10^-16^ | 0.1883 | 1.30x10^-6^ | 1.07x10^-24^ | 0.0897 | 0.2206 | 1.13x10^-8^ |

**Supplementary Table 7 footnote.** Results are standardised beta coefficients, 95% confidence intervals, and p-values. Models are multivariable linear regression models with raw CMR metrics set as the response (outcome) variable and vascular risk factors or cardiovascular diseases entered as exposure variables. For the vascular risk factor models, covariates are: age, sex, ethnicity, deprivation, education, body mass index, physical activity, hypertension, high cholesterol, diabetes and smoking. For the prevalent cardiovascular disease models, there is additional adjustment for atrial fibrillation, stroke, IHD, and MI. *indicates p-values significant with a false discovery rate of 0.05, giving an approximate threshold of 0.025. AF: atrial fibrillation, CMR: cardiovascular magnetic resonance, GLS: global longitudinal strain, i: indicates indexation to body surface area, IHD: ischaemic heart disease, LAEF: left atrial ejection fraction, LAV: maximum left atrial volume, LVEDV: left ventricular end-diastolic volume, LVEF: left ventricular ejection fraction, LVGFI: left ventricular global function index, LVM: left ventricular mass, MI: myocardial infarction.

**Supplementary Table 8. Associations of mutually adjusted CMR metrics with incident cardiovascular disease and mortality outcomes in Cox proportional hazard models with full confounder adjustment**

| **CMR Metric** | **AF** | **Stroke** | **IHD** | **MI** | **All-cause mortality** | **CVD mortality** |
| --- | --- | --- | --- | --- | --- | --- |
| LAVi (ml/m^2^) | 1.47* | 1.13 | 1.09 | 1.06 | 1.11 | 1.34* |
|  | [1.28, 1.70] | [0.98, 1.30] | [1.01, 1.19] | [0.93, 1.21] | [1.00, 1.23] | [1.05, 1.71] |
|  | 8.89x10^-8^ | 0.0837 | 0.0318 | 0.4037 | 0.0429 | 0.0188 |
| LAEF (%) | 0.64* | 0.83* | 0.87* | 0.87* | 0.96 | 0.85 |
|  | [0.56, 0.73] | [0.73, 0.95] | [0.81, 0.95] | [0.76, 0.98] | [0.87, 1.06] | [0.69, 1.05] |
|  | 3.32x10^-11^ | 0.0055 | 8.65x10^-4^ | 0.0278 | 0.4239 | 0.1306 |
| LVM: LVEDV | 1.07 | 1.23* | 1.29* | 1.14 | 1.12 | 1.11 |
| (g/m^2^) | [0.93, 1.25] | [1.07, 1.43] | [1.18, 1.40] | [0.99, 1.31] | [1.00, 1.25] | [0.86, 1.43] |
|  | 0.3440 | 0.0048 | 3.58x10^-9^ | 0.0723 | 0.0405 | 0.4358 |
| LVEF (%) | 0.93 | 0.94 | 0.93 | 0.98 | 0.87* | 0.66* |
|  | [0.81, 1.06] | [0.82, 1.08] | [0.86, 1.01] | [0.86, 1.13] | [0.79, 0.96] | [0.54, 0.80] |
|  | 0.2673 | 0.3648 | 0.1041 | 0.8239 | 0.0066 | 3.59x10^-5^ |
| GLS (%) | 0.97 | 1.11 | 1.08 | 1.05 | 1.13* | 1.08 |
|  | [0.84, 1.12] | [0.96, 1.28] | [0.99, 1.18] | [0.91, 1.21] | [1.02, 1.26] | [0.86, 1.37] |
|  | 0.7111 | 0.1699 | 0.0773 | 0.4968 | 0.0217 | 0.5063 |

**Supplementary Table 8 footnote.** Results are hazard ratios, 95% confidence intervals, and p-values. Covariates are: LAV, LAEF, LVM/LVEDV, GLS, GLFI, age, sex, ethnicity, deprivation, education, body mass index, hypertension, high cholesterol, diabetes, physical activity, smoking. The CMR variables are principal component analysis rotated variables. *indicates statistically significant p-values with a false discovery rate of 0.05, giving an approximate threshold of 0.028 for this table. AF: atrial fibrillation, CMR: cardiovascular magnetic resonance, CVD: cardiovascular disease; LVEF: left ventricular ejection fraction, GLS: global longitudinal strain, i: indicates indexation to body surface area, IHD: ischaemic heart disease, LAEF: left atrial ejection fraction, LAV: maximum left atrial volume, LVEDV: left ventricular end-diastolic volume, LVM: left ventricular mass, MI: myocardial infarction.

**Supplementary Table 9. Associations of raw individually entered CMR metrics with incident CVD and mortality outcomes in Cox proportional hazard models with full confounder adjustment**

| **CMR Metric** | **AF** | **Stroke** | **IHD** | **MI** | **All-cause mortality** | **CVD mortality** |
| --- | --- | --- | --- | --- | --- | --- |
| LAVi (ml/m^2^) | 1.72* | 1.17* | 1.09 | 1.08 | 1.11 | 1.49* |
|  | [1.49, 1.99] | [1.02, 1.35] | [1.00, 1.18] | [0.95, 1.23] | [1.00, 1.22] | [1.17, 1.89] |
|  | 5.90x10^-14^ | 0.0285 | 0.0492 | 0.2462 | 0.0469 | 0.0011 |
| LAEF (%) | 0.59* | 0.79* | 0.84* | 0.85* | 0.89* | 0.69* |
|  | [0.52, 0.66] | [0.69, 0.89] | [0.78, 0.91] | [0.75, 0.96] | [0.81, 0.98] | [0.57, 0.83] |
|  | 9.16x10^-20^ | 1.59x10^-4^ | 8.05x10^-6^ | 0.0107 | 0.0180 | 1.36x10^-4^ |
| LVM/LVEDV | 1.00 | 1.21* | 1.27* | 1.13 | 1.09 | 0.99 |
| (g/ml) | [0.86, 1.17] | [1.05, 1.40] | [1.16, 1.37] | [0.98, 1.30] | [0.98, 1.22] | [0.76, 1.29] |
|  | 0.9932 | 0.0107 | 2.52x10^-8^ | 0.0834 | 0.1161 | 0.9298 |
| LVEF (%) | 0.84* | 0.90 | 0.92 | 0.96 | 0.85* | 0.62* |
|  | [0.73, 0.96] | [0.78, 1.03] | [0.84, 1.00] | [0.84, 1.10] | [0.77, 0.94] | [0.52, 0.74] |
|  | 0.0094 | 0.1306 | 0.0450 | 0.5539 | 0.0013 | 2.69x10^-7^ |
| LVGFI (%) | 0.83* | 0.82* | 0.82* | 0.90 | 0.82* | 0.56* |
|  | [0.71, 0.97] | [0.70, 0.95] | [0.75, 0.90] | [0.78, 1.05] | [0.73, 0.91] | [0.44, 0.71] |
|  | 0.0193 | 0.0107 | 2.81x10^-5^ | 0.1890 | 3.90x10^-4^ | 1.69x10^-6^ |
| GLS (%) | 1.15 | 1.19* | 1.16* | 1.10 | 1.20* | 1.42* |
|  | [0.99, 1.33] | [1.03, 1.38] | [1.06, 1.27] | [0.96, 1.27] | [1.07, 1.33] | [1.12, 1.80] |
|  | 0.0658 | 0.0155 | 7.62x10^-4^ | 0.1698 | 0.0010 | 0.0043 |

**Supplementary Table 9**. Results are hazard ratios, 95% confidence intervals, and p-values estimating the association of each CMR metrics with the corresponding incident CVD and mortality outcomes in Cox hazard proportional models, with each cell representing a separate model. Covariates are: age, sex, ethnicity, deprivation, education, body mass index, physical activity, hypertension, high cholesterol, diabetes, smoking. CMR metrics are entered individually in raw form. *indicates p-values significant with a false discovery rate of 0.05, giving an approximate threshold of 0.03 for this table. AF: atrial fibrillation, CMR: cardiovascular magnetic resonance, CVD: cardiovascular disease, GLS: global longitudinal strain, i: indicates indexation to body surface area, IHD: ischaemic heart disease, LAEF: left atrial ejection fraction, LAV: maximum left atrial volume, LVEDV: left ventricular end-diastolic volume, LVGFI: left ventricular global function index, LVM: left ventricular mass, MI: myocardial infarction.

**Supplementary Table 10. Number of incident events observed in the whole cohort and selected subsets**

|  | AF | Stroke | IHD | MI | All-cause mortality | CVD mortality |
| --- | --- | --- | --- | --- | --- | --- |
| Whole sample; n=25896 | 180 | 178 | 530 | 197 | 331 | 58 |
| Healthy cohort (no CVD, no VRF); n=12393 | 51 | 60 | 141 | 47 | 109 | 16 |
| Participants with VRF, but no CVD, n=11237 | 93 | 88 | 348 | 115 | 161 | 28 |
| Participants with CVD; n=2266 | 36 | 30 | 41 | 35 | 61 | 14 |

**Supplementary Table 10.** AF: atrial fibrillation; CVD: cardiovascular disease; IHD: ischaemic heart disease; MI: myocardial infarction; VRF: vascular risk factor.
