# Supplementary material for: Left atrial structure and function are associated with cardiovascular outcomes independent of left ventricular measures: a UK Biobank CMR study
Source: Eur Heart J Cardiovasc Imaging. Author manuscript; Available in PMC 2022 Aug 22. (PMC9365306; doi:10.1093/ehjci/jeab266)
Supplement: Supplementary data [file EMS151232-supplement-Supplementary_data.pdf]

Downloaded from https://academic.oup.com/ehjcimaging/advance-article/doi/10.1093/ehjci/jeab266/6462016 by Birmingham-Southern College user on 20 July 2022

Supplementary data

Supplementary data are available at *European Heart Journal - Cardiovascular Imaging* online.
